# High Throughput Sequencing Identifies Misregulated Genes in the *Drosophila* Polypyrimidine Tract-Binding Protein (*hephaestus*) Mutant Defective in Spermatogenesis

**DOI:** 10.1371/journal.pone.0150768

**Published:** 2016-03-04

**Authors:** Vinod Sridharan, Joseph Heimiller, Mark D. Robida, Ravinder Singh

**Affiliations:** Department of Molecular, Cellular and Developmental Biology, University of Colorado at Boulder, Boulder, Colorado, United States of America; University of Valencia, SPAIN

## Abstract

The *Drosophila* polypyrimidine tract-binding protein (dmPTB or *hephaestus*) plays an important role during spermatogenesis. The *heph*^*2*^ mutation in this gene results in a specific defect in spermatogenesis, causing aberrant spermatid individualization and male sterility. However, the array of molecular defects in the mutant remains uncharacterized. Using an unbiased high throughput sequencing approach, we have identified transcripts that are misregulated in this mutant. Aberrant transcripts show altered expression levels, exon skipping, and alternative 5’ ends. We independently verified these findings by reverse-transcription and polymerase chain reaction (RT-PCR) analysis. Our analysis shows misregulation of transcripts that have been connected to spermatogenesis, including components of the actomyosin cytoskeletal apparatus. We show, for example, that the *Myosin light chain 1* (*Mlc1)* transcript is aberrantly spliced. Furthermore, bioinformatics analysis reveals that *Mlc1* contains a high affinity binding site(s) for dmPTB and that the site is conserved in many *Drosophila* species. We discuss that *Mlc1* and other components of the actomyosin cytoskeletal apparatus offer important molecular links between the loss of dmPTB function and the observed developmental defect in spermatogenesis. This study provides the first comprehensive list of genes misregulated *in vivo* in the *heph*^*2*^ mutant in *Drosophila* and offers insight into the role of dmPTB during spermatogenesis.

## Introduction

Heterogeneous nuclear ribonucleoproteins (hnRNPs) are ubiquitously expressed and associate with primary transcripts. One of these RNA-binding proteins, the polypyrimidine-tract-binding protein (PTB), which is also referred to as hnRNP I, binds to polypyrimidine tracts. These binding sites contain UCUU and UUCU sequence motifs [1–5]. PTB has been linked to regulation of mRNA splicing, polyadenylation, translation, mRNA stability/degradation, and mRNA localization (reviewed in [6–9]). Of the three human PTB genes (PTBP1, PTBP2, and PTBP3), PTBP1 is more widely expressed, and PTBP2 and PTBP3 show expression restricted to specific tissues [10].

There is one PTB homolog in *Drosophila melanogaster*, which is also known as *hephaestus* (*heph*). Varied phenotypes are associated with *Drosophila heph* mutants: embryonic lethality, sensory bristle and wing margin abnormalities [4, 11–13]. During wing development and embryogenesis, it has been implicated in modulating Notch signaling [11, 14–20]. During oogenesis, *heph* has been linked to *oskar* mRNA translational repression [21] and Grk signaling in the female germline [22].

We reported previously that a major *heph* or *dmPTB* transcript is expressed specifically in the male germline and that it correlates with male fertility in *Drosophila* [13]. Spermatogenesis, which is a complex process, shows remarkable cytological similarities between *Drosophila* and mammals: germ cell maintenance, mitotic divisions preceding meiosis, and morphological changes involving all cellular components (reviewed in [23]). Extensive genetic analysis of spermatogenesis has revealed multiple mutations that exhibit critical cytological blocks, for example, stem cell renewal, mitotic and meiotic amplification, and spermatid differentiation [23, 24]. In fact, *heph* was first identified in a genetic screen for male sterility [24]. Nonetheless, how relevant mutations influence specific stages during spermatogenesis had been a mystery. We showed that the dm*PTB* loss of function *heph*^*2*^ mutant disrupts the cellular process of spermatid individualization, leading to male sterility [4]. Spermatid individualization represents the terminal step in spermatogenesis.

To understand the sex-specific biological role of dmPTB in male sterility, the question arises what are specific mRNAs that are misregulated in the *heph*^*2*^ mutant? We undertook an unbiased high throughput sequencing approach to investigate the role of *heph* in *Drosophila* at a genome-wide level. We used RNA-Seq analysis of mRNAs from wild type and *heph*^*2*^ mutant flies and show that loss of *heph* function results in misregulation of several specific transcripts, including mRNA splicing misregulation, that may provide a missing link(s) to its role in spermatogenesis.

## Results

### High throughput sequence analysis for transcriptome profiling

The molecular basis of the developmental defects in the *heph*^*2*^ mutant is currently unknown. Therefore, we used high throughput sequencing to identify transcripts that are misregulated in *heph*^*2*^ mutant flies. We performed the RNA-Seq protocol on polyadenylated RNA from wild-type control and *heph*^*2*^ mutant adult flies. We obtained 57,738,593 sequence reads for the wild-type control and 44,791,181 sequence reads for the *heph*^*2*^ mutant. Using TopHat [25], over 90% of the sequences could be mapped to the *Drosophila* genome and about 7% corresponded to splice junction reads (“Table 1”).

**Table 1:** Sequencing and mapping statistics for the *heph*^*2*^ analysis.

Group	# of reads	# of spliced reads	% mapped to genome
*heph2/TM3* adult males(control)	57,738,593	3,981,756 (7%)	91%
*heph2/heph2* adult males	44,791,181	3,154,576 (7%)	92%

**Fig 1:**
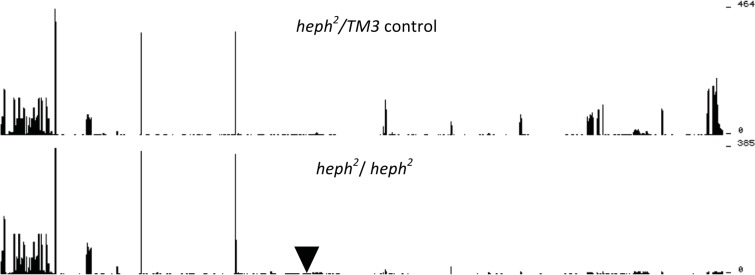
Loss of RNA-Seq coverage in the *heph*^*2*^ homozygous mutant in the 3’ portion of the *heph* gene, downstream of the *P* element insertion site. The control sample is shown on the top graph and the *heph*^*2*^ homozygous mutant on the bottom graph. The inverted triangle represents the location of the *P* element insertion in an intron (intron has been condensed to save space). There are two transcription start sites for the *dmPTB or heph* locus, producing transcripts with proximal and distal 5’UTRs. The abundant male germline-specific transcript, which is disrupted in the *heph*^*2*^ mutant, results from the use of the distal or upstream promoter. The *P* element in *heph*^*2*^ is inserted into an intron corresponding to the transcript with distal 5’ UTR. However, for the *heph*^*03429*^ mutant, showing embryonic lethality, the *P* element is inserted into an intron downstream of the proximal 5’ transcript start site. This transcript start site is relevant for the non-sex-specific, including embryonic, function of *dmPTB*; no reads were found that spanned the other end of the *P* element insertion into the *heph* gene [26].

The *heph*^2^ mutant was generated via *P* element transposon mutagenesis [24]. Multiple sequence reads from the *heph*^2^ mutant confirmed the insertion of the transposon *P* element in the *heph* locus. As shown in Fig 1, the *heph*^*2*^ mutation disrupted synthesis of *dmPTB* transcripts in *heph*^*2*^ homozygotes—a severe drop-off in mapped reads downstream of the *P* element insertion, which is located within a *heph* intron. These results show that the *heph*^*2*^ mutation resulted in truncation of about 96% of the *dmPTB* transcripts. Considering full-length transcripts, the *heph*^*2*^ homozygous mutants have roughly 4% of wild-type mRNA levels. Thus, transcriptome profiling establishes for the first time how *dmPTB* transcripts are disrupted in the *heph*^*2*^ mutant.

### Transcriptome profiling identifies mRNA expression level differences

**Fig 2:**
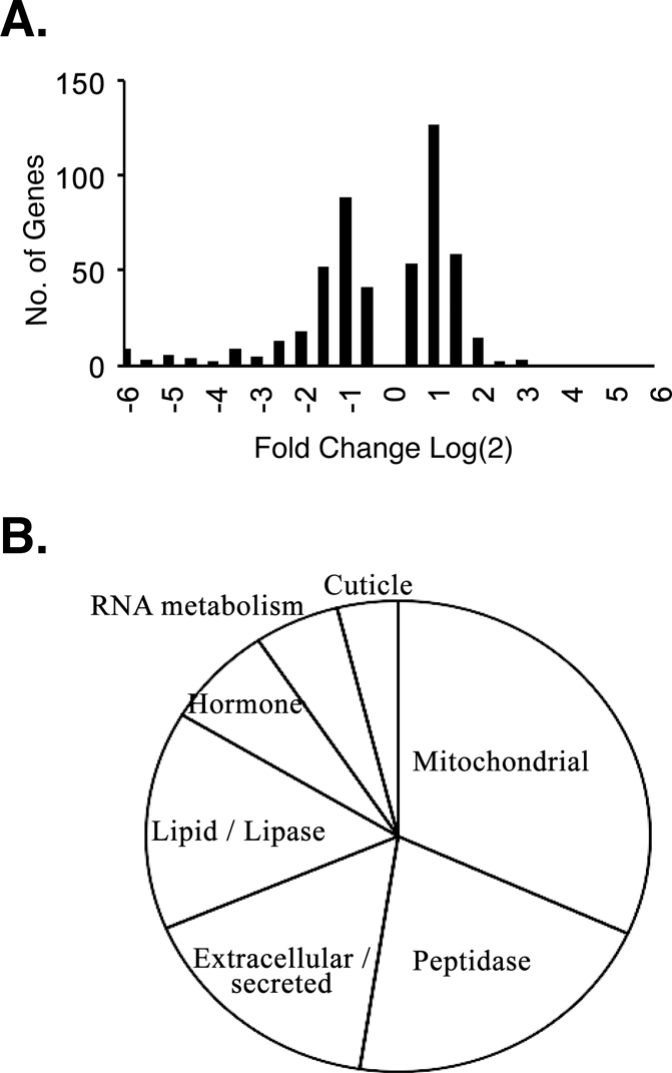
Effect of the *heph*^*2*^ mutation on fold-changes in gene expression. Histogram of fold-changes in the *heph*^*2*^ mutants of genes called significant by Cufflinks (note that Cufflinks did not call any genes significant below 0.78 log_2_ change). The list is provided in S1 Table. (B). Pie chart representing gene ontology categories over-represented in the genes that are significantly affected in the *heph*^*2*^ mutant.

We compared the two samples for expression levels of individual genes. In the *heph*^*2*^ dataset, the Cufflinks software identified 493 genes as differentially expressed (Fig 2A, see Methods for details). There were numerous genes whose expression level changed over 2-fold (Fig 2A). Next, we asked if some functional gene ontology categories were over-represented in these differentially expressed genes. Using gene ontology analysis with DAVID (The Database for Annotation, Visualization and Integrated Discovery), we subjected to comparative analysis the above gene set that showed difference in expression levels between the wild-type control and the *heph*^2^ mutant. Our analysis showed several over-expressed gene categories such as RNA metabolism, extracellular/secreted, hormones, and peptidases (Fig 2B and Table 2). Given that many genes play multiple cellular roles, further studies will be needed to establish whether and how the GO terms are related to the spermatogenesis phenotype. When we carefully inspected these genes, there were two differentially expressed genes that had been known to be involved in spermatogenesis: *Nc* (*Nedd2-like caspase* or *Dronc*) and *oxen* (*ox*) [24, 27, 28]. In the *heph*^*2*^ mutant, *Dronc/Nc* levels were reduced to about 50% of wild-type levels, and *ox* levels were increased by 2.5 times compared to wild type (see Discussion for the functional relevance of these genes). Since *heph*^*2*^ mutants cause male but not female sterility, we also compared genes differentially expressed in *heph*^*2*^ to those expressed mostly in the male germline, but not in the female germline, using modEncode RNA Seq data [29]. The intersection of the male-specific genes (genes that were significantly higher in the adult male germline vs. the female germline) and the genes expressed in *heph*^*2*^ yielded 185 genes (about 86 genes were expected by chance). None of these genes, however, was related to spermatid individualization based on prior annotation. Our results show significant changes in gene expression of specific genes in the *heph*^2^ mutant.

**Table 2:** Selected gene ontology categories from the *heph*^*2*^ analysis.

GO category	DAVID—*P* value(Benjamini-adjusted)	Up or down-regulation
extracellular / secreted	1 x 10^−15^	up/down
mitochondrial	2 x 10^−14^	up/down
peptidase	1 x 10^−8^	up/down
stress response	1 x 10^−8^	up
signal peptide	3 x 10^−6^	down
lipase	3 x 10^−5^	up/down

The p-values for ontology groups are shown from the DAVID analysis.

### Transcriptome profiling identifies misregulated mRNA isoforms

Differences in mRNA isoforms could result from use of alternative transcription start sites (TSS), alternative 5’ splice sites, alternative 3’ splice sites, exon skipping, and alternative 3’ ends. Alternative isoform candidates in the *heph*^*2*^ dataset were first identified using MISO (Mixture of ISOforms) [30]. While MISO called 35 alternative isoform candidates as significant, we found, upon manual inspection, that most of these candidates appeared to be weak, and thus were not pursued. Our experience revealed the limitation of this algorithm. However, during our inspection we observed significant isoform differences in several categories. Table 3 shows a list of the filtered candidates and observed fold-changes. Transcripts such as *Paramyosin* (*Prm*) and *Tequila* were examples of alternative 5’ untranslated regions resulting from alternative transcription start sites (5.3 fold). CG43293 was an example of alternative 5’ splice site (significant fold change: 27% vs. 0%). *Myosin alkali light chain 1* (*Mlc1*), *CG1674*, and *Aldolase* (*Ald*) were examples of alternative exons (2–4 fold). The transcripts above showed the most significant isoform differences in the *heph*^2^ mutant. We conclude that the *heph*^*2*^ mutation results in misregulation of specific mRNA isoforms.

**Table 3:** *heph*^*2*^ differentially expressed isoforms.

Gene	Alternative Isoform	Fold-change	Gene ontology
*Paramyosin* (*Prm*)	TSS	5.3	Structural constituent of muscle
*Tequila*	TSS	5.3	Serine-type endopeptidase
*Myosin alkali light chain 1* (*Mlc1*)	Exon	2.1	Actin motor
*CG1674*	Exon	4.1	Unknown
*Aldolase* (*Ald*)	Exon	1.8	Fructose-bisphosphate aldolase
*CG43293*	5’ SS	Large	Unknown

The candidates were manually filtered. The fold change was calculated based on values from control (%) versus *heph*^*2*^ mutant (%), calculated from exon-exon spanning ‘spliced’ reads supporting each isoform. The Bayes factor/odds ratio for these candidate as called by MISO was 10^9^ or better.

### RT-PCR analysis confirms expression level differences

**Fig 3:**
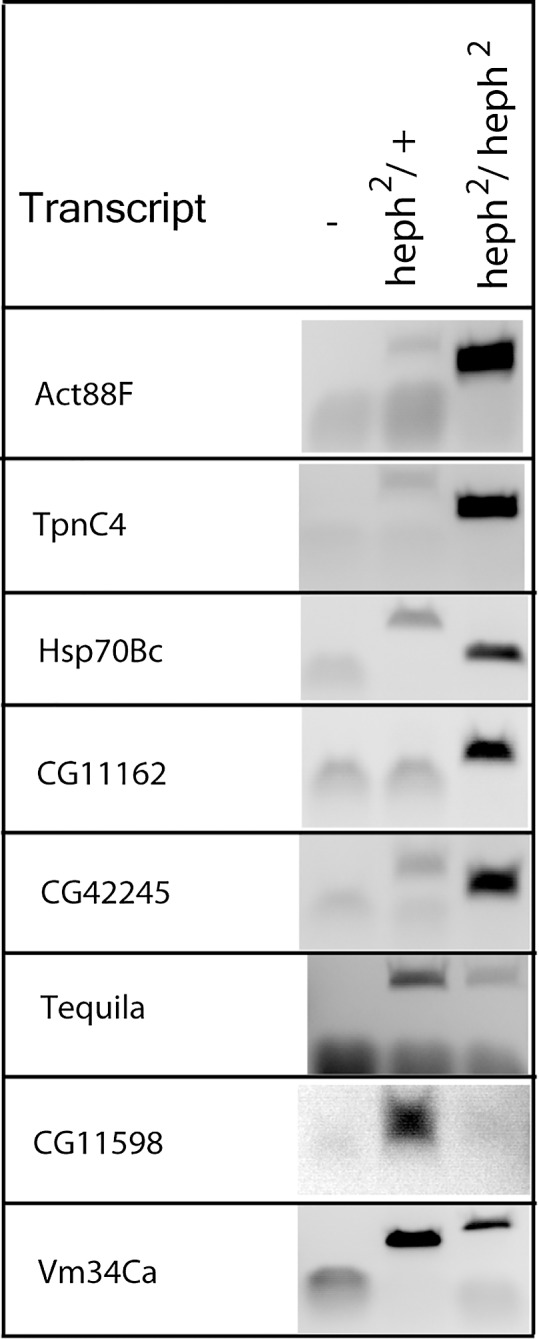
RT-PCR analysis reveals differentially expressed transcripts. RNA from the wild type control and the *heph*^*2*^ mutant was analyzed by RT-PCR analysis. Gene-specific primer pairs were used for amplification of several randomly picked genes that showed significant differential expression. For *Tequila*, primer pairs were designed to test alternative 5’ ends (transcription start site usage).

We randomly selected several candidates from specific gene ontology categories for analysis by RT-PCR for their expression level differences between the wild-type control and the *heph*^2^ mutant. Fig 3 shows that RT-PCR analysis recapitulated the up and down regulation observed from the Illumina sequencing reads. For example, transcripts such as *Act88F*, *TpnC4*, *Hsp70Bc*, *CG11162*, and *CG42245* were significantly upregulated in the *heph*^2^ mutant. On the other hand, transcripts such as *CG11598* and *Vm34Ca* exhibited downregulation (Table 4 and S1 Table). We conclude that RT-PCR and high throughput sequencing independently confirm misregulation of specific transcripts in the *heph*^2^ mutant.

**Table 4:** Fold changes for selected candidates (ratio of RNA-Seq reads—mutant versus control).

Gene	Fold-change	Up or Down regulation
*Act88F*	+4.6	Up
*TpnC4*	+5.2	Up
*Hsp70Bc*	+10.5	Up
*CG11162*	+20.1	Up
*CG42245*	+79.8	Up
*Tequila*	-1.45	Down
*CG11598*	-32.1	Down
*Vm34Ca*	-88.1	Down

### RNA analysis confirms misregulated isoforms

**Fig 4:**
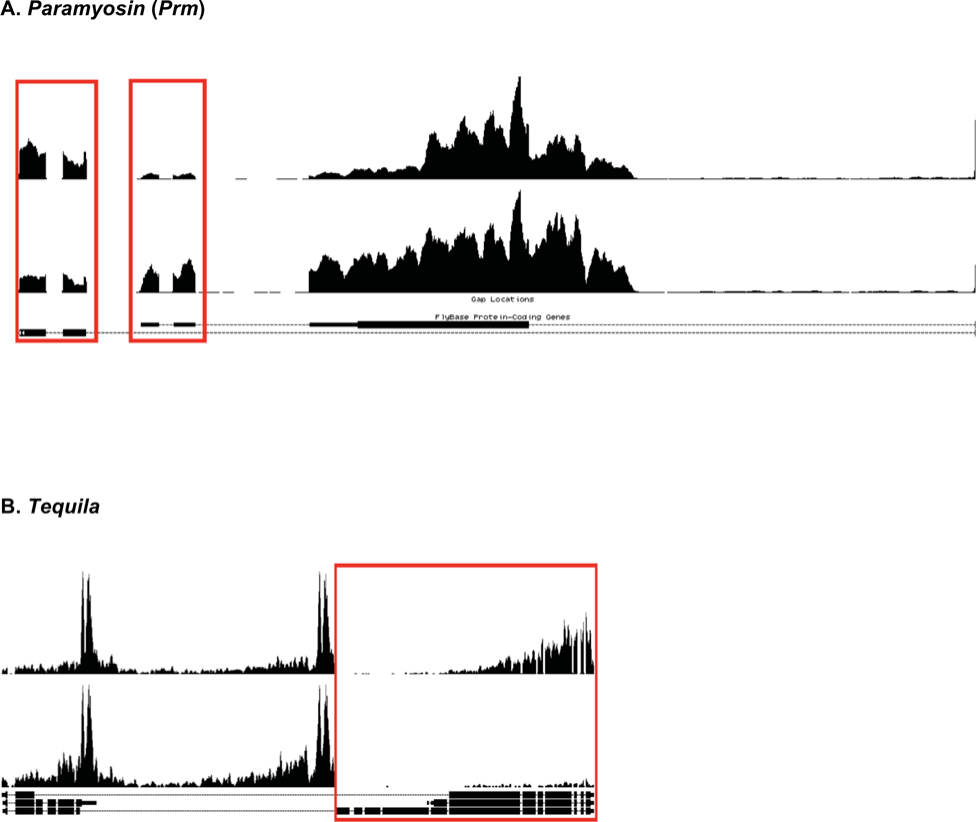
Use of alternative transcription start sites in the *heph*^*2*^ mutant. (**A)** RNA Seq read pileup across a *Paramyosin* alternative transcription start site, where alternative transcription start sites highlighted by the box. (**B)** RNA Seq read pileup across a *Tequila* alternative transcription start site, where the 5’ end showing the most difference is highlighted by the box. For RT-PCR validation of *Tequila* alternative transcription start site usage, see Fig 3.

**Fig 5:**
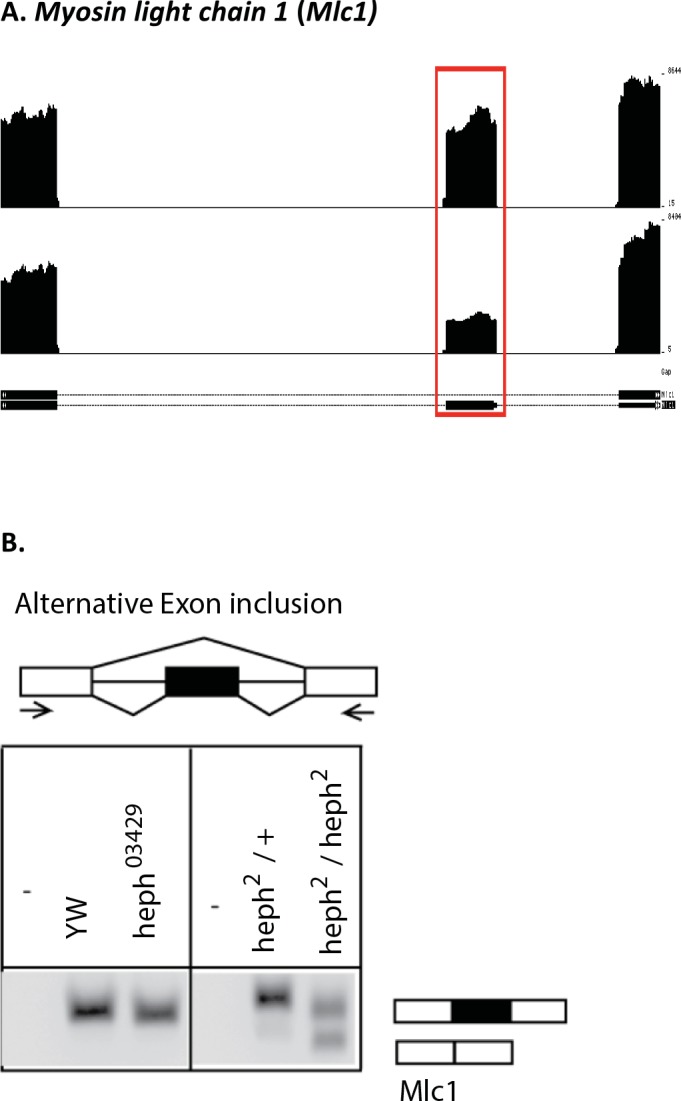
Alternative splicing of the *Mlc1* gene in the *heph*^*2*^ mutant. RNA Seq pileups were generated using UCSC Genome Browser, y-axis is auto-scaled to show differences in isoform fractions, alternative exon is highlighted by the box. **(A)** RNA-Seq read pileup across the *Mlc1* skipped exon in control (top) and *heph*^*2*^ mutant (bottom) flies, showing significantly increased exon skipped in the *heph*^*2*^ mutant. (**B)** RT-PCR analysis of *Mlc1* for exon skipping, using primers in the flanking exons shown by the arrows.

Next, we analyzed the *Prm* and *Tequila* transcripts for their isoform differences. Indeed, RT-PCR analysis showed the presence of alternative isoforms for the *Tequila* and *Prm* genes (Figs 3 and 4A and 4B; data not shown), validating the analysis of high throughput sequencing. *Myosin light chain 1* (*Mlc1*) was an example of altered splicing pattern in the *heph*^2^ mutant (Fig 5A). We analyzed *Mlc1* expression pattern by RT-PCR, and found that it recapitulated the quantitative difference in the *Mlc1* skipped exon in the mutant (Fig 5B). We also analyzed embryonic mRNA from another embryonic lethal allele *heph*^03429^, which did not produce exon skipping. Thus, the altered splicing pattern observed here was specific to the *heph*^2^ mutation that causes male sterility. We also excised the two bands from the gel and confirmed exon inclusion and exclusion in *Mlc1* by direct sequencing. These findings show that loss of *dmPTB* function in the *heph*^2^ mutant results in significant differences in mRNA isoforms for specific transcripts.

### A PTB binding site is phylogenetically conserved in Mlc1

**Fig 6:**
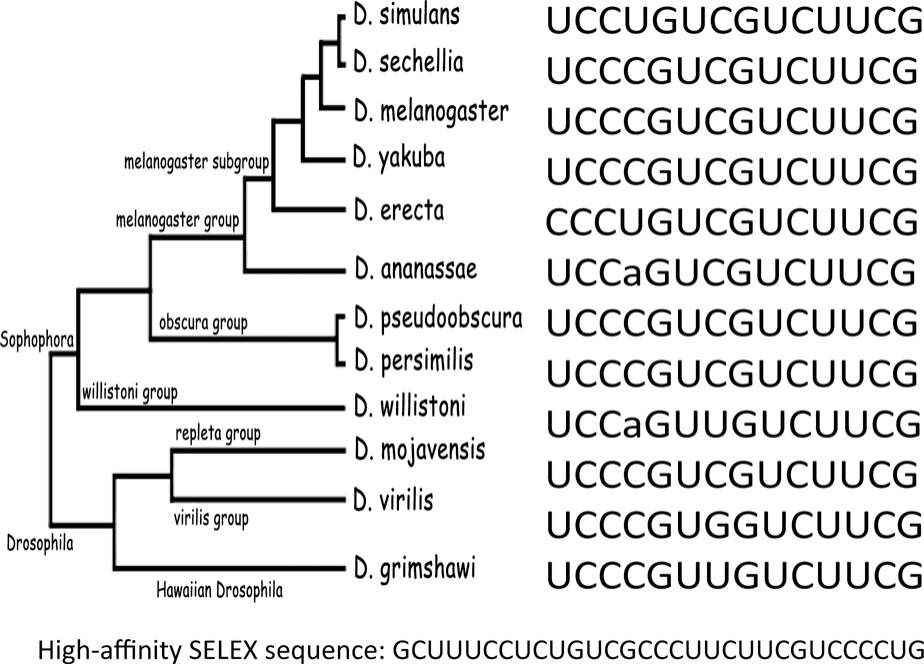
Identification of a conserved PTB binding site in *Mlc1*. Putative PTB binding site in *Mlc1* conserved in 12 Drosophila species is shown. The highest affinity SELEX sequence that binds PTB *in vitro* is shown for comparison.

We previously showed that the *heph*^*2*^ mutation disrupted spermatid individualization, an actomyosin cytoskeletal-related process [4, 31]. Thus, *Mlc1* was an attractive candidate for which misregulation could contribute to the observed male sterility phenotype of the *heph*^*2*^ mutant. To determine if there was a dmPTB binding site in this transcript we analyzed sequences in the vicinity of the altered exon use in *Mlc1* from 12 *Drosophila* species. We found that the skipped *Mlc1* exon contained an extended C/U-rich site and that this site was conserved in 12 different Drosophila species (Fig 6). In fact, this C/U-rich sequence is very similar to the PTB consensus binding site obtained through the SELEX enrichment of RNAs that bound PTB with high affinity [4, 5]. The expression of other myosin light chains in *Drosophila* including *Androcam*, *Calmodulin*, *Mlc2*, and *Mlc-c* was not significantly affected in our analysis of the *heph*^*2*^ mutant. We conclude that the *Mlc1* transcript contains a conserved high affinity PTB binding site.

## Discussion

We used a high throughput sequencing approach for transcriptome profiling and have identified transcripts that are misregulated in the *heph*^*2*^ mutant. These include transcripts that show differences in expression levels (up or down regulation) and mRNA isoforms (with alternative 5’ ends or exons) and some of them have been linked to spermatogenesis. The candidates revealed by the genome-wide analysis have been independently validated for their misregulation. Finally, phylogenetic comparison among *Drosophila* species reveals a conserved high affinity PTB binding site near a misregulated splice site in the *Mlc1* gene. These findings are consistent with the idea that the regulatory RNA binding protein dmPTB likely affects many molecular functions *in vivo*.

We chose to study the *heph*^*2*^ allele because it shows a specific developmental defect during spermatogenesis, leading to male sterility. In contrast to the sex-specific expression of the major *dmPTB* transcript in *Drosophila* [13], the human PTB is ubiquitously expressed. Our high throughput analysis shows that in the *heph*^*2*^ mutant *dmPTB* reads are absent past the site of transposon insertion. This lack of *dmPTB* expression manifests in significant misregulation of several specific downstream genes.

Among the misregulated transcripts revealed here, several are excellent candidates for their role in the spermatogenesis process, and thus provide connections to how loss of dmPTB function can lead to known spermatogenesis defects or some other male-specific defect that remains to be characterized. For example, *Dronc/Nc*, which is misregulated in the *heph*^*2*^ mutant, has been previously shown to be involved in one of the caspase activation pathways necessary for spermatid individualization [28]. This previous study elegantly showed that multiple caspases and caspase regulators, involving ARK- and HID- dependent activation of DRONC at sites of spermatid individualization, are required for this non-apoptotic process of spermatid individualization. Similarly, *oxen*, another misregulated transcript in the *heph*^*2*^ mutant, was previously uncovered in a genetic screen that was designed to identify male-sterile mutants [24]. In fact, this same genetic screen also revealed the *heph*^*2*^ mutant. Thus, our observation is consistent with a possible functional relationship between *heph* and *oxen*. We argue that a quantitative differences in *Dronc/Nc* and *ox* transcripts in the *heph*^*2*^ mutant are biologically relevant because, based on our previous observation, quantitative reduction in dmPTB levels correlates well with the severity of the male-sterile phenotype [4]. These observations on *Dronc/Nc* and *ox* misregulation strongly suggest regulatory relationships, acting in the same developmental pathway–spermatogenesis.

We previously showed that adult *heph*^*2*^ testes contain all spermatogenesis stages up to the elongated spermatids but no motile sperm [13] and accumulate cysts of elongated spermatids with numerous bulges along their length [4], which is characteristic of defects in the spermatid individualization process [32]. In fact, when we specifically examined, with DAPI or phalloidin staining, the integrity of the individualization complex (IC), which is characterized by a tightly cross-linked actin cytoskeletal cone, this complex was disrupted in the *heph*^*2*^ mutant [4]. Given that this terminal step of spermatogenesis involves an actomyosin cytoskeletal apparatus or directional F-actin regulation, the question arises what components or regulators of this actomyosin cyskeletal apparatus are involved. To our knowledge, these candidates have not been experimentally shown to display male sterile defects. We speculate that *Act88F*, *Prm*, *TpnC4*, and/or *Myosin light chain 1*, which have emerged as candidates for regulation by dmPTB in our genome-wide screen, are likely important players in this process and their misregulation could in part account for male sterility in general and the disrupted IC in particular. In regards to other misregulated genes reported here, although UCUU and UUCU sequence motifs [3] can be found in most genes, we have been unable to find an obvious high affinity PTB binding similar to the one shown in Fig 6 [4, 5]. It is an open question whether these genes are further downstream or contain a functional PTB-binding site that remains to be identified. Future studies should distinguish between these possibilities. Our analysis has also revealed misregulated genes that have not been linked to spermatogenesis yet.

We note that all of the alternative isoform candidates were confirmed to be expressed in the testis using RNA-Seq data from the modEncode project [29]; the testis isoform expression pattern matched with that of the *heph*^*2*^*/TM3* control and not the *heph*^*2*^ homozygotes. We also compared the differentially expressed genes in *heph*^*2*^ and *heph*^*03429*^. There was little overlap, beyond the number of genes that would be expected by chance, between the differentially expressed genes in the *heph*^*2*^ and *heph*^*03429*^ analyses (S1 Fig). The *heph*^*03429*^ mutation was previously linked to misregulation of *Notch* in embryos [11, 17–19]. While Notch plays a role in spermatogenesis [33], whether it functions during spermatid individualization, which is defective in the *heph*^*2*^ mutant [4], remains to be established.

A prominent role of sex-specific RNA expression and splicing has been extensively studied in *Drosophila melanogaster*, where a series of alternative splicing events (*Sxl* → *tra* → *dsx*) leads to somatic sexual differentiation [34]. In *Caenorhabditis elegans*, a cascade of regulatory events or repression at the level of transcription or translation mediates sexual differentiation [35]. In mammals, including mice and humans, the key regulator gene *SRY* on the Y chromosome leads to testes differentiation, which produces testosterone and masculinizes all somatic tissues. In fact, the *dsx* ortholog is conserved in *C*. *elegans* (*mab-3*) and humans (*DMRT*) [36, 37]. Recent, high throughput analysis clearly shows abundance of sex-specific RNA expression differences in *Drosophila* and humans, including pre-gonadal tissues such as brain [38–43]. Recognition of sex-specific RNA differences in humans and vertebrates has important implications in normal development and clinical aspects of various diseases such as neurological disorders that show sex-biased disease development, pathology, and recovery (reviewed in [44]). While sex-specific alternative mRNA processing is well established in *Drosophila melanogaster*, environmental, genetic, and inherited epigenetic mechanisms (chromatin modifications and remodeling), non-coding RNA, and RNA and DNA editing are emerging as important additional regulatory players in sex-specific gene expression, leading to sexual dimorphism in mammals and vertebrates.

In addition to RNA-Seq [26] and the phylogenetic sequence analysis used here, several other approaches have been employed in other studies of RNA-protein interaction: yeast-three-hybrid assay [45]; CLIP [46]; Fast-FIND (*Fast*-*F*ully *I*ndexed *N*ucleotide *D*atabase) [47]; PAR-CliP (Photoactivatable-Ribonucleoside-Enhanced Crosslinking and Immunoprecipitation) [48]; and RIP-Seq (or RIP-ChIP). As noted above, the short, degenerate (pyrimidine-rich sequence) PTB binding site appears frequently in the genome and makes identification of RNA-protein interactions that are functionally relevant even more difficult. Our analysis has provided a list of candidates for future functional analysis and a useful dataset that can be combined with other genome-wide studies to obtain a more complete list of misregulated transcripts. Follow-up molecular, genetic, and biochemical analysis should provide better understanding of the molecular basis for spermatogenesis.

## Materials and Methods

### Fly stocks and RNA preparation

The *heph*^*2*^ stock was obtained from the Bloomington Stock Center. Flies were raised on standard cornmeal food at 17–25°C. Poly(A)^+^ RNA from 1 microgram total RNA was obtained from the *heph*^*2*^ mutant and wild-type control (*heph*^*2*^ */ TM3*) whole adult homozygous *heph*^*2*^ males aged 1–3 days after eclosion, and mRNA-seq sequencing libraries (standard Illumina TruSeq) were prepared according to manufacturer’s instructions (Illumina, Inc., San Diego CA, USA). The *heph*^*2*^ mutant and wild-type control libraries were sequenced on an Illumina HiSeq 2000 sequencer (singleton 50 base-pair reads).

### RNA Seq: alternative isoform analysis

TopHat 2.0.4 was used to map reads to the Flybase *Drosophila melanogaster* r5.44 genome annotation (GTF and Fasta files), with the—no-novel-juncs and—microexon-search parameters (mapping statistics are shown in Table 1) [25]. Alternative splicing analysis was first attempted using the MISO [30] software package using event-specific analysis (as opposed to whole-isoform analysis). Alternative splicing candidates were called using an isoform fraction difference threshold of 0.2 and a minimum Bayes Factor of 10 (at least 10-to-1 odds, or 0.0909 probability, that the alternative event call was not due to chance alone). Most of the MISO alternative isoform candidates, however, showed little observable difference upon manual inspection of the reads across alternative junctions and so were not pursued. Cufflinks did not call any alternative splicing or alternative transcription start sites as significant, and so was not used for isoform-specific analysis [49]. We also used an alternative isoform algorithm which called ratios of exon-exon spanning reads supporting each isoform, using the probability mass function of the binomial distribution to estimate a p-value (which was then converted into a log-odds value). De novo prediction of new splice sites using TopHat did not yield any significant differential isoforms, so only annotated splice junctions were used in this analysis. Traces were generated using the UCSC genome browser/tools. For additional methods details on RNA-Seq expression and isoform analysis, see [26].

### RNA Seq: differential gene expression analysis

Cufflinks version 2.0.0 was used to determine significant overall gene expression in the *heph*^2^ mutant analyses. Cufflinks did not call any gene significant under an absolute fold-change value of 0.781 in the *heph*^*2*^ analysis.

### Search for PTB sites

Putative PTB sites were searched through the UCSC 15 species insect alignment, which includes 12 Drosophila species (ftp://hgdownload.cse.ucsc.edu/goldenPath/dm3/multiz15way/). A PTB binding site was defined as a stretch of at least nine C or U residues allowing at most one G, conserved within 25 nucleotides (not including indels) in at least 9 species in the 15-species genomic alignment. Conserved PTB sites were also searched manually around alternative splice sites in the UCSC genome browser.

### RT-PCR validation of candidates

Alternative splicing candidates were validated using RT-PCR primers that spanned the alternative transcriptional area, using one PCR primer set to span both alternative isoforms to yield two product sizes. Alternative transcription site candidates were validated using an identical reverse primer and separate forward primers. RpS15 (a ribosomal protein gene) RT-PCR was used as a loading control with primers designed over a constitutive intron boundary to rule out the presence of genomic DNA. PCR primers used are shown in S1 Table.

### Gene Ontology analysis

The Database for Annotation, Visualization and Integrated Discovery (DAVID) v6.7 was used for gene ontology analysis online. The pie chart (in Fig 2) represents the genes in only the most overrepresented ontology categories.

## Supporting Information

S1 Fig A comparison of sequence reads from the *heph*^*2*^ and *heph*^*03429*^ mutants and primer sequences are provided. (PDF)

S1 Table Analysis of sequence reads between control and *heph*^*2*^. (XLSX)
