# Supplementary material for: High Throughput Sequencing Identifies Misregulated Genes in the Drosophila Polypyrimidine Tract-Binding Protein (hephaestus) Mutant Defective in Spermatogenesis
Source: PLoS One. 2016 Mar 4;11(3):e0150768. doi: 10.1371/journal.pone.0150768 (PMC4778870; doi:10.1371/journal.pone.0150768)
Supplement: S1 Fig — (PDF) [file pone.0150768.s001.pdf]

Supplementary Online Material:

We compared the *heph*$^{2}$ and *heph*$^{03429}$ mutants for gene expression profiles. There was little overlap between the differentially expressed genes in the *heph*$^{2}$ and *heph*$^{03429}$ analyses. There were 63 differentially expressed genes shared between the two analyses (Fig. S1A), which is about the number of genes that would be expected by chance. Further, none of the alternative isoform candidates overlapped. This possibility reflects how the two mutants result in developmental phenotypes – embryonic phenotype and adult phenotypes in testes. Nonetheless, the more general gene ontology comparison of differentially expressed genes showed some similarity, for example, the profile of stress response genes, extracellular and signal peptide-related genes, and peptidase genes (Fig. S1B). Further experiments will be needed to understand the biological significance of these observations.

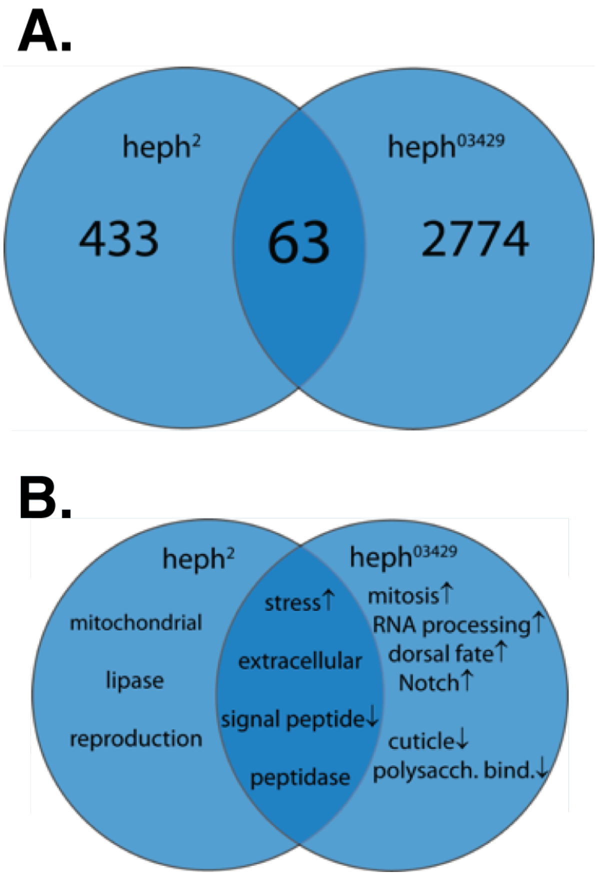


**Figure S1. A)** Venn diagram of the number of differentially expressed genes in the *heph*$^{2}$ and *heph*$^{03429}$ analyses. There are 63 differentially expressed genes in common between the *heph*$^{2}$ and *heph*$^{03429}$ analyses, changing in the same direction. **B)** Venn diagram of overlapping gene ontologies. Arrows denote whether genes in ontology group were mostly up or down-regulated. If no arrow, the genes in the group were mixed.

**Supplementary Table 1. Primers used for validation of differential expression in the *heph*$^{2}$ mutant.**

| Gene / isoform | Forward primer | Reverse primer |
|---|---|---|
| RpS15 | TCTACAACGGCAAGGACTTCGG | CGTTTCATTTATTCACGCATCA |
| Mlc1 | CCCATCTACTCACAGGTCAAGAAG | ATGGTTGTCGCTAAAAGTTTGTTG |
| Tequila 3' end | GCAGAATGTCCATCCTAAGTTGTG | ATCCAAGTAATCATGTGCAGAGAG |
| Tequila 5' end | TTCCCATTTTGGCCGATCACGTAT | AGCAAGCTCTCATTGATTTTCTCG |
| Prm | CGTCTGCACGAATTGGAACTGGAG | CATTGAGCTGCTCGATCTCGATCG |
| Act88 | CGCACAACAATTATCATCCAACTC | CACGACTCTTACGATTAGTTCTTC |
| Vm34Ca | ATAAGGAGGATAAGATGCTCGGCT | TTGATCAGGGGTTAGTGAAGTCTG |
| CG11598 | TCCTGAGGAACTTGATCAACTACA | CTCCTTTGCTGTCGAACTATCATC |
| CG11162 | CGCAGTCCGGGGATTATCTG | GGGTGAAGATGCCCGCATAG |
| Hsp70bc | AGGAGATGAGCACGGGCAAG | CCTGCTCCACGGACTGCTTT |
| Tpnc4 | GCAAACTGGCAGCTCGCTTT | TACCGGATCCATCCGCATCA |
| CG42245 | CCGCAGGACCAGGAGGACTA | CGTCAGATCCGCTCGCTTTT |
